# Pandemic Information Dissemination and Its Associations With the Symptoms of Mental Distress During the COVID-19 Pandemic: Cross-sectional Study

**DOI:** 10.2196/28239

**Published:** 2021-12-03

**Authors:** Ole Myklebust Amundsen, Asle Hoffart, Sverre Urnes Johnson, Omid V Ebrahimi

**Affiliations:** 1 Department of Clinical Psychology University of Bergen Bergen Norway; 2 Modum Bad Psychiatric Hospital Vikersund Norway; 3 Department of Psychology University of Oslo Oslo Norway

**Keywords:** information sources, COVID-19, avoidance, psychopathology

## Abstract

**Background:**

The 2020-2021 COVID-19 pandemic has added to the mental health strain on individuals and groups across the world in a variety of ways. Viral mitigation protocols and viral spread affect people on all continents every day, but at widely different degrees. To understand more about the mental health consequences of the pandemic, it is important to investigate whether or how people gather pandemic-related information and how obtaining this information differentially affects individuals.

**Objective:**

This study aimed to investigate whether and to what extent higher levels of COVID-19–related media consumption across information sources are associated with the symptoms of anxiety, health anxiety, and depression, and whether and to what extent using social media and online interactive platforms versus traditional media platforms is associated with the symptoms of anxiety, health anxiety, and depression. Additionally, we aimed to investigate whether and to what extent avoidance of COVID-19–related information is associated with the aforementioned symptoms.

**Methods:**

In a cross-sectional preregistered survey, 4936 participants responded between June 22 and July 13, 2020. Eligible participants were adults currently residing in Norway and were thus subjected to identical viral mitigation protocols. This sample was representative of the Norwegian population after utilizing an iterative raking algorithm to conduct poststratification. As 2 subgroups (transgender and intersex individuals) were too small to be analyzed, the final sample for descriptive statistics and regressions included 4921 participants. Multiple regressions were used to investigate associations between the symptoms of psychopathology and COVID-19–related information dissemination. Part correlations were calculated as measures of the effect size for each predictor variable. Due to the large anticipated sample size, the preregistered criterion for significance was set at *P*<.01.

**Results:**

The symptoms of anxiety and health anxiety were significantly associated with obtaining information from newspapers (*P*<.001), social media (*P*<.001), and the broader categories of online interactive (*P*<.001) and traditional media (*P*<.001). The symptoms of depression were significantly associated with obtaining information from newspapers (*P*=.003), social media (*P*=.009), and the broader category of online interactive media (*P*<.001). Additionally, avoidance of COVID-19–related information showed a significant association in all 3 domains of psychopathological symptoms (anxiety and depression, *P*<.001; health anxiety, *P*=.007).

**Conclusions:**

This study found significant associations between the symptoms of psychopathology and the use of media for obtaining information related to the COVID-19 pandemic. Significant findings for obtaining information through newspapers, social media, and online interactive media were seen across all 3 measures of psychopathology. Avoidance of COVID-19–related information and associations with the symptoms of psychopathology emerged as core findings, with generally higher effect sizes compared with information attainment.

**Trial Registration:**

ClinicalTrials.gov NCT04442360; https://clinicaltrials.gov/ct2/show/NCT04442360

## Introduction

### Background

As of September 2021, the COVID-19 (SARS-CoV-2) pandemic has led to more than 4.5 million deaths worldwide and more than 218 million confirmed cases [1]. While governments strive to provide their inhabitants with up-to-date and accurate information, deliberate and unintentional misinformation has spread across the world [2]. Efficacious information delivery is critical to inform inhabitants of national, regional, and local viral mitigation protocols (VMPs) that are implemented in lieu of a vaccine. Access to mental health services has become an important issue during protracted pandemic restrictions and VMPs [3], and the psychological consequences of the pandemic have been elaborated upon, but its possible long-term consequences are not yet known [4,5].

Imperative pandemic-related information needs to be distributed appropriately to societal members. Concurrently, scientists and government officials have become increasingly concerned with the mental health consequences of VMPs, such as lockdowns, curfew, and more generally forced social isolation, during the COVID-19 pandemic [6]. The scientific community has urged the importance of investigating how high exposure to health messaging disseminated across divergent media platforms is related to mental health. There are indications of increased psychopathological symptoms when attempting to mitigate physical disease and viral spread [6-9].

Although information distribution and access are crucial to inform citizens about changing VMPs, steps to increase quality of life despite restrictions and information on how to live life under lockdown and restriction would be beneficial. However, the world is currently experiencing an overabundance of information, which is referred to as an *infodemic* by the World Health Organization to highlight that there is an ongoing information pandemic parallel to the viral pandemic. Earlier research has shown that overly negative news may lead to aversion and consequently to avoidance of news [10].

In the current COVID-19 pandemic, the level of contagion of many countries has risen sharply over time, or has risen and fallen in waves. Additionally, news has been dominated by high levels of contagion and death, and has been repeated incessantly on national and international news channels. Some research has also shown that individuals have a propensity toward passing along negative news even if the news is exaggerated or there is more positive news that is equally relevant at hand, and the current global infodemic may partly be made up of the spread of such frequently repeated negative news [11].

Studies from previous pandemics (eg, Zika, swine flu, and Ebola) and the current pandemic have revealed that individuals highly engaged in information-seeking behavior tend to become more focused on the threatening aspects of infectious diseases, experiencing increased distress and anxiety [12,13]. This is especially so when individuals perceive themselves as having little control over the threat [13].

Several years of research have given credence to the idea that increased consumption of information from social media in general can be detrimental to psychological health [14-19], and that increased use of such media is associated with the symptoms of both anxiety and depression [17,20-22]. Social media (eg, Twitter, Facebook, TikTok, Snapchat, Instagram, and WeChat), characterized by fast-paced information streams, are often not curated or validated for facts [23] and have in recent years been an arena for the spread of misinformation [24]. In the current pandemic, social media have been used to dispense information more rapidly to the population [25].

Information is also actively spread through other platforms, such as television, newspapers, websites, and online forums. Additionally, individuals are also informed about the pandemic face-to-face via their peers. News distributed on television can be especially volatile. A study found that the median airtime for medical news stories was just 33 seconds, and these stories did not cite the origin of the information they provided and did not convey recommendations to the public [26]. The association of consumption of information obtained via traditional media with mental distress has also been investigated, but the interaction is less clear than it is for social media [27].

Furthermore, the avoidance of news can have direct consequences for individuals and the society at large. In the current infodemic, news avoidance may be a consequence of news overload (ie, unintentional avoidance). During the current pandemic, relevant advice from intergovernmental, national, and regional authorities has been constantly changing, putting a large strain on individuals who want to keep themselves updated on the current recommendations for hygienic behavior and social distancing. An overload of information sources from platforms, such as newspapers and television, may cause those avoiding these platforms to seek pandemic-relevant information from other sources, such as friends and family, which has been found to be related to decreased adherence to social distancing protocols [28]. Thus, understanding and combating avoidance of information, specifically from official sources, is crucial.

Avoiding information can entail not being confronted with uncertainty. In crises, such as the current pandemic, large groups of people are required to live life with a far higher degree of everyday uncertainty than usual [29]. Individuals with high intolerance of uncertainty attempt to reduce this uncertainty through behavioral control, exemplified as checking the internet and other sources for information [29-31]. Furthermore, health anxiety is tied to an increase in the search for health-related information, with psychological distress as a consequence [32]. With an increase in the tendency to search for information, there is also an increase in different media that provide the information, with potentially differential effects.

Thus, it is crucial to investigate how dissemination of divergent sources of information is related to mental health symptoms in the general population during the present COVID-19 pandemic and parallel infodemic.

### Research Questions and Hypotheses

The following research questions and hypotheses were investigated, as presented in the preregistered protocol of the study:

1. Research questions: Is there a differential effect among different information sources on health anxiety, depression, and anxiety? To what extent and how are different information sources related to the symptoms of health anxiety, depression, and anxiety?

2. Hypothesis 1: Media consumption across all information sources will significantly be associated with depression and anxiety symptoms, with increased media consumption in general associated with higher levels of health anxiety, depression, and general anxiety.

3. Hypothesis 2: Using social media and online interactive platforms (ie, forums and blogs) to obtain news about the pandemic in comparison to using traditional media (ie, television, radio, and newspapers) will be associated with higher levels of general anxiety, depression, and health anxiety. Actively staying away from information will further significantly be associated with higher levels of anxiety, depression, and health anxiety.

## Methods

### Overview

This cross-sectional study is part of The Norwegian COVID-19 Mental Health and Adherence Project, and utilizes data collected in the second stage of data collection in this project. This study investigated the association of various sources of information acquisition concerning COVID-19 with psychopathology, specifically the symptoms of anxiety, depression, and health anxiety during the COVID-19 pandemic. Following the guidelines of Strengthening the Reporting of Observational Studies in Epidemiology [33], and the health estimate reporting standards laid out in the GATHER statement [34], this study was designed and preregistered prior to any data collection. The preregistered protocol can be found at ClinicalTrials.gov (NCT04442360). All components of the submitted study adhere to its preregistered protocol.

### Study Design, Participants, Procedure, and Timing

The data for this cross-sectional study were collected via an online survey between June 22 and July 13, 2020, and involved the second stage of data collection for the project. In the first wave of data collection, a survey was distributed on national, regional, and local information platforms (ie, television, radio, and newspapers), in addition to dissemination to a random selection of Norwegian adults through a Facebook Business algorithm. Details regarding procedure and timing can be found elsewhere [9]. In the first stage, data were obtained from 10,061 participants. For the second stage of data collection, all participants who had provided informed consent to participate further were invited to take part. The eligible participants were adults (ie, aged 18 years or above) currently residing in Norway, who were thus subject to identical VMPs. A total of 4936 participants made up the sample for this study. The survey was administered approximately 1 week after major VMPs were lifted in Norway and included a period when the national VMPs and guidelines did not shift. Additionally, no novel information was provided by the Norwegian government regarding social distancing protocols during this period.

### Measurements

#### Demographic Characteristics

Information regarding participant age, sex, education level, ethnic background, and regional affiliation level were collected.

#### Symptoms of Psychopathology

The Generalized Anxiety Disorder-7 (GAD-7) [35] is a scale for identifying the level of anxiety. It consists of 7 items measuring anxiety on a 4-point Likert scale (range 0-3), with the total score ranging from 0 to 21. For GAD-7, internal consistency was good, with a Cronbach α of .90.

The Patient Health Questionnaire-9 (PHQ-9) [36] is a measure of depression severity that consists of 9 items scored on a 4-point Likert scale (range 0-3), with the total score ranging from 0 to 27. The internal consistency of this scale was excellent in this sample, with a Cronbach α of .91.

The symptoms of health anxiety were measured with 2 items from the validated Health Anxiety Inventory [37] and 2 items adapted for the COVID-19 pandemic, with one item measuring the specific fear of being infected by the coronavirus and another item measuring the fear of dying from the coronavirus on a 4-point Likert scale (range 0-3). Internal consistency was acceptable for these health anxiety–related questions, with a Cronbach α of .77.

#### Information-Seeking Behavior

Participants were asked to estimate the amount of time they had spent obtaining information regarding the COVID-19 pandemic using various media platforms since the beginning of March 2020, which was the onset of pandemic restrictions in Norway. This included (1) recognized newspapers; (2) television channels; (3) social media; (4) forums, blogs, podcasts, and other online outlets (excluding online newspapers); (5) friends, family, and acquaintances; and (6) other sources. Moreover, active avoidance of COVID-19–related information on all media was measured. These variables were measured on an 8-point Likert scale (range 0-7; 0=never, 7=multiple times per hour). Two new variables were created from the existing media variables. Using online interactive media was defined as using social media, forums, and blogs, and was consequently the sum of these variables. Using traditional media was defined as using television and newspapers, and consisted of the sum of these variables.

### Statistical Analyses

The descriptive analyses of the present data were reported using means and standard deviations. Two educational groups were collapsed due to a low N value, and these groups were also used in multiple regressions. For hypothesis 1, 3 separate multiple regression analyses with the symptoms of anxiety, depression, and health anxiety as criterion variables were conducted. The predictor variables were the extents of information obtained about the COVID-19 pandemic from (1) recognized newspapers; (2) television channels; (3) social media; (4) forums, blogs, podcasts, and other online outlets (excluding online newspapers); (5) friends, family, and acquaintances; and (6) other sources, as well as actively staying away from information. Additionally, we controlled for age, gender, education level, and the presence of a psychiatric diagnosis.

For hypothesis 2, 3 separate multiple regression analyses were conducted, with the symptoms of anxiety, depression, and health anxiety as criterion variables. Media variables were collapsed as described above into traditional media and online interactive media. Additionally, we controlled for age, gender, education level, and the presence of a psychiatric diagnosis.

For all multiple regressions, part correlations were calculated. Part correlation, also referred to as semipartial correlation, is a measure low in bias that is easily interpretable. Part correlations are estimates of the strength of a predictive relationship and can be interpreted as an effect size measure using the Cohen criteria of small effect size *>*0.10, medium effect size *>*0.30, and large effect size *>*0.50 [38,39]. Standard criteria for multicollinearity were fulfilled [40] in all multiple regression models, and all assumptions were met.

The preregistered criterion for significance was set at *P<*.01, given the anticipated sample size. All statistical analyses were conducted using R (version 4.0.3; The R Project for Statistical Computing).

### Poststratification Weights

In this study, the sampled gender, age, education, regional affiliation, and ethnic background deviated somewhat from the population parameters. All these deviations (minor deviations as well as larger deviations) were weighted and adjusted to accurately reflect the Norwegian adult population. More weight was assigned to underrepresented units and less weight to overrepresented units. Weights were calculated using the R packages “anesrake” (version 0.8) and “survey” (version 4.0). These packages use an iterative algorithm (ie, raking ratio estimation) to iteratively assign appropriate weights to each subgroup by turn to avoid the distribution matching of one factor skewing the distribution of other factors. The weights outputted as a result of this algorithm were applied to the data set, resulting in a weighted data set with parameters closely matching that of the true population and yielding a highly representative sample. All statistical analyses utilized this weighted representative sample.

## Results

### Sample Characteristics

A total of 4936 individuals were included in this sample, but as 2 of the subgroups (transgendered and intersex individuals) were too small to be factors in our analyses, a sample of 4921 individuals was used for descriptive statistics (Table 1) and for all multiple regression analyses. Table 1 also shows the weighted N values after poststratification with the population parameters as a reference point. Means and standard deviations for the media variables and avoidance can be found in Multimedia Appendix 1, showing that mean time spent gathering information about COVID-19 was the highest for newspapers, television, and social media. A correlation matrix for media variables can be found in Multimedia Appendix 2, showing a negative correlation of avoidance with all media variables as expected.

**Table 1:** Sample characteristics and weighted characteristics (N=4921).

Variable		Sample, n (%)	Weighted, n (%)	Actual population, %
**Sex**				
	Female	3911 (79.48)	2427 (49.32)	49.77
	Male	1010 (20.52)	2494 (50.68)	50.23
**Age group (years)**				
	18-30	1703 (34.61)	1069 (21.73)	23.22
	31-44	1606 (32.64)	1340 (27.24)	24.30
	45-64	1344 (27.31)	1707 (34.70)	31.26
	65 or above	268 (5.44)	804 (16.34)	21.22
**Education**				
	Junior high school^a^	191 (3.88)	573 (11.64)	25.40
	Completed high school	736 (14.96)	1931 (39.25)	37.00
	Currently studying	775 (15.75)	409 (8.32)	6.70
	Completed university degree	3219 (65.41)	2006 (40.78)	30.90
**Ethnic background**				
	Native Norwegian	4563 (92.73)	4346 (88.32)	85.29
	European	274 (5.57)	401 (8.16)	7.58
	Asian	39 (0.79)	117 (2.38)	4.56
	African	6 (0.12)	18 (0.37)	1.85
	North America/Oceania	15 (0.30)	17 (0.35)	0.27
	South/Middle/Latin-America	24 (0.49)	21 (0.43)	0.45
**Regional affiliation**				
	East Norway	3103 (63.06)	2966 (60.28)	58.32
	West Norway	1162 (23.61)	922 (18.74)	20.28
	Mid-Norway	482 (9.79)	781 (15.87)	15.95
	Northern Norway	174 (3.54)	251 (5.11)	5.45

^a^This category is collapsed and consists of individuals who did and those who did not complete junior high school.

### Information-Seeking Behavior and the Symptoms of Anxiety

The multiple regression model examining the factors associated with the symptoms of anxiety can be found in Table 2 and Multimedia Appendix 3. Multimedia Appendix 3 displays the regression results for the variables online interactive media and traditional media, explaining 33% of the variance in the data. Gender was a significant predictor of anxiety, with female gender being associated with higher levels of anxiety symptoms. Age was also a significant predictor of anxiety symptoms, with lower age being associated with higher symptoms of anxiety. Additionally, having a pre-existing mental health condition was associated with increased anxiety symptoms. Time spent obtaining information about the pandemic using newspapers was a significant predictor of anxiety symptoms, and more time spent reading newspapers was associated with a higher degree of anxiety symptoms.

**Table 2:** Predictors of anxiety symptoms in the weighted representative sample (N=4921; adjusted R^2^=0.33).

Variable	Beta	SE of beta	*P* value	Part correlation (r)
Intercept	5.16	0.39	<.001	1.00
Gender^a^	−0.59	0.18	.001	−0.06
Age	−0.06	0.01	<.001	−0.18
Education	−0.19	0.09	.03	−0.04
Mental health condition	4.34	0.29	<.001	0.37
Newspapers	0.28	0.07	<.001	0.08
Television	0.01	0.07	.86	0.00
Social media	0.24	0.07	<.001	0.08
Forums and blogs	0.14	0.09	.10	0.04
Friends and family	−0.06	0.10	.58	−0.01
Other	0.04	0.08	.57	0.01
Avoidance	0.36	0.08	<.001	0.10

^a^Female=0; male=1.

Time spent obtaining information from social media was a significant predictor of anxiety symptoms, with more time spent being associated with a higher degree of anxiety symptoms. Obtaining information using both traditional media and online interactive media was a significant predictor of anxiety, and the relationship was positive. Finally, avoiding information about COVID-19 entirely was a significant predictor of anxiety symptoms, with a higher degree of avoidance being associated with an increased level of anxiety symptoms.

Among these significant associations, part correlations revealed the factors most strongly associated with the symptoms of anxiety while accounting for all other variables. The most prominent factors associated with the symptoms of anxiety included having a pre-existing mental health condition (part correlation=0.37), age (part correlation=−0.18), obtaining information from online interactive media (part correlation=0.10), and avoiding information about the pandemic (part correlation=0.10), all revealing small to medium effect sizes. Lower effect sizes emerged for obtaining information concerning the COVID-19 pandemic from social media (part correlation=0.08), for obtaining information from newspapers (part correlation=0.08), and generally for traditional media (part correlation=0.08) and the effect of gender (part correlation=−0.06).

Obtaining information about the pandemic from television, forums and blogs, family and friends, and other sources was unrelated to the symptoms of anxiety. Education level was also unrelated to the symptoms of anxiety at our significance level.

### Information-Seeking Behavior and the Symptoms of Depression

The multiple regression model examining the factors associated with depressive symptoms is depicted in Table 3 and Multimedia Appendix 4. Multimedia Appendix 4 displays the regression results for the variables online interactive media and traditional media, explaining 36% of the variance in the data. Age, gender, and education level were significant predictors of depressive symptoms, where lower age, lower education level, and a trend toward female gender were associated with a higher level of depressive symptoms. Having a pre-existing mental health condition was associated with a greater level of depressive symptoms. Time spent obtaining information about the pandemic from newspapers and social media was a significant predictor of depressive symptoms, with more time spent reading newspapers being associated with a higher level of depressive symptoms.

**Table 3:** Predictors of depressive symptoms in the weighted representative sample (N=4921; adjusted R^2^=0.33).

Variable	Beta	SE of beta	*P* value	Part correlation (r)
Intercept	8.42	0.54	<.001	1.00
Gender^a^	−0.68	0.25	.005	−0.05
Age	−0.07	0.01	<.001	−0.16
Education	−0.34	0.12	.004	−0.05
Mental health condition	6.41	0.39	<.001	0.41
Newspapers	0.27	0.09	.002	0.06
Television	−0.12	0.09	.19	−0.03
Social media	0.22	0.08	.009	0.05
Forums and blogs	0.18	0.11	.11	0.03
Friends and family	−0.25	0.13	.06	−0.04
Other	−0.02	0.10	.82	0.001
Avoidance	0.64	0.11	<.001	0.13

^a^Female=0; male=1.

Obtaining information about the pandemic through online interactive media was a significant predictor of depressive symptoms, where more time spent was associated with a higher level of depressive symptoms. Avoiding information entirely was a significant predictor of depressive symptoms, where increasing avoidance of COVID-19–related information was associated with a higher level of depressive symptoms.

Among these significant associations, part correlations revealed the factors most strongly associated with depressive symptoms while accounting for all other variables. The strongest factors associated with depressive symptoms included having a pre-existing mental health condition (part correlation=0.41), age (part correlation=−0.16), and avoiding information about COVID-19 (part correlation=0.13), all revealing small to medium effect sizes. Smaller effect sizes were observed for education level (part correlation=−0.06), and obtaining news about the pandemic through newspapers (part correlation=0.06), online interactive media (part correlation=0.08), and traditional media (part correlation=0.03).

Obtaining information about COVID-19 from television, forums and blogs, family and friends, and other sources was unrelated to depressive symptoms. Obtaining information from traditional media was also unrelated to depressive symptoms.

### Information-Seeking Behavior and the Symptoms of Health Anxiety

The multiple regression model examining the factors associated with the symptoms of health anxiety can be found in Table 4 and Multimedia Appendix 5. Multimedia Appendix 5 displays the regression results for the variables online interactive media and traditional media, and the regressions in Table 4 and Multimedia Appendix 5 explain 16% and 15% of the variance in the data, respectively. Education was a significant predictor of health anxiety symptoms, with a lower education level being associated with a higher level of health anxiety symptoms. Having a pre-existing mental health diagnosis was a significant predictor of health anxiety symptoms.

**Table 4:** Predictors of health anxiety symptoms in the weighted representative sample (N=4921; adjusted R^2^=0.16).

Variable	Beta	SE of beta	*P* value	Part correlation (r)
Intercept	0.52	0.18	.003	1.00
Gender^a^	−0.13	0.09	.13	−0.03
Age	0.00	0.00	.13	0.03
Education	−0.19	0.04	<.001	−0.09
Mental health condition	1.19	0.14	<.001	0.24
Newspapers	0.20	0.03	<.001	0.13
Television	0.02	0.03	.61	0.01
Social media	0.12	0.03	<.001	0.09
Forums and blogs	0.03	0.05	.49	0.02
Friends and family	−0.03	0.05	.61	−0.01
Other	0.02	0.04	.69	0.01
Avoidance	0.09	0.04	.007	0.06

^a^Female=0; male=1.

Time spent obtaining information from newspapers was a significant predictor of health anxiety symptoms, with more time spent being associated with an increased level of health anxiety symptoms. Time spent obtaining information from social media was a significant predictor of health anxiety symptoms, with more time spent being associated with a higher level of health anxiety symptoms. Obtaining information about the pandemic from both traditional media and online interactive media was a significant predictor of health anxiety, where more time spent on these media was associated with increased symptoms. Avoiding information entirely was a significant predictor of health anxiety symptoms, with higher levels of avoidance being positively associated with health anxiety symptoms.

Among these significant associations, part correlations revealed the factors most strongly associated with the symptoms of health anxiety while accounting for all other variables. The strongest factors associated with the symptoms of health anxiety included having a pre-existing mental health condition (part correlation=0.23), using traditional media to obtain information about the pandemic (part correlation=0.14), and time spent gathering information from newspapers (part correlation=0.13), in addition to using online interactive media in general (part correlation=0.10), revealing small effect sizes. Obtaining information through social media (part correlation=0.09), education level (part correlation=−0.09), and avoiding news about COVID-19 (part correlation=0.06) had smaller effect sizes.

Age; gender; and information attainment related to COVID-19 from television, family and friends, forums and blogs, and other sources were unrelated to the symptoms of health anxiety.

## Discussion

### Hypothesized Effects

This study investigated the association of various sources of pandemic information dissemination with psychopathology during the COVID-19 pandemic in a Norwegian sample. For hypothesis 1, the strongest effect sizes were found for using social media and newspapers to obtain information about COVID-19. Support was not found for the hypothesized associations of obtaining information from television, forums and blogs, friends and family, and other sources with the symptoms of anxiety, health anxiety, and depression.

For hypothesis 2, using either online interactive media or traditional media had the strongest effect sizes pertaining to the symptoms of psychopathology, except for the measure of depressive symptoms, where using traditional media was not statistically significant. Additionally, as hypothesized, avoiding news about COVID-19 was significantly associated with the symptoms of anxiety, health anxiety, and depression.

### Information-Seeking Behavior and the Symptoms of Psychopathology

Our findings seem to be in line with previous pandemic research results, where the use of different sources of information about COVID-19 was associated with increased symptoms of anxiety, depression, and health anxiety [12,13,41,42]. During such pandemics, individuals are required to live life with a certain degree of uncertainty due to the novelty of the situation [29], such as living with the risk of getting infected, not knowing if others you interact with are contagious, and being constantly wary of potential situations that could put one at risk.

Individuals with high intolerance to uncertainty may attempt to reduce this uncertainty through behaviors, such as checking the internet (ie, social media, forums, and blogs) and other sources (ie, newspapers and television) for information [29-31], exuding internal control over an external problem. Furthermore, health anxiety is tied to an increase in the search for health-related information, with psychological distress as a potential consequence [32]. With an increased tendency to search for information, there is also an increase in different media providing that information, with potentially differential effects. Additionally, using social media in general is associated with the symptoms of mental distress [14-19].

As with social media, other unverified platforms have been investigated in previous pandemics. During the 2009 swine flu outbreak, language in blogs mentioning the swine flu was more dramatic compared to a control, and this language later spread to newspapers [43], indicating that monitoring of multiple types of media may result in exposure to similar dramatic language over time across different media. Interestingly, a study by Blakey [44] during the 2015 Zika outbreak found that knowledge of the virus predicted anxiety, indicating that it may not only be dramatic language that has a detrimental effect, but also general knowledge about the outbreak itself, an important point that national and regional governments need to take into consideration when preparing information for their citizens.

### Information Avoidance and the Symptoms of Psychopathology

In line with the findings of this investigation, avoidance in anxiety literature is empirically documented as a maladaptive behavior that maintains anxiety levels [45-50]. Our results indicate that the association of news avoidance with anxiety and depression was greater than the association of the use of different media platforms for information about the pandemic with the symptoms of psychopathology, when viewing part correlations as effect sizes.

One reason for this might be that individuals who explicitly avoid news show general tendencies of avoidance, although existing mental health conditions were controlled for in our analyses. Previous research has shown that those who avoid news intentionally are exposed to news regardless [51-53]. Actively avoiding news and then being exposed to the news regardless might trigger the very feelings one seeks to avoid, although in an uncontrolled context, it might be more hurtful than being exposed to the news in the first place. Avoiding news might be maladaptive in the same sense that avoiding a feared stimuli for a person who has an anxiety disorder hinders recovery. Avoiding such information can shield the individual from information that might trigger existing fearful thoughts, but can possibly increase the span of attentiveness toward general negative experience, which may in turn be reinterpreted as threatening symptom stimuli through attentional biases [54].

Consequently, a core question is how we can reach those avoiding pandemic-related information, as these individuals seem to be at increased risk of experiencing psychopathological symptoms. To better understand the reasons underlying avoidance, future studies should explore whether pandemic-related news avoidance is intentional or unintentional, and how it is related to “news overload.” Skovgaard and Andersen [55] argue that one potential cause for avoidance is the overload of information, which is in accordance with the term infodemic. This connection is further elucidated in recent studies on social media [56,57], showing that news avoidance directly affects news efficacy.

Furthermore, individuals may be engaged in intentional avoidance due to low trust in news and the negativity of news [55]. The authors propose that these problems underlying intentional avoidance may be solved by providing clear and concise information, avoiding presentation of opinions, sticking to reporting facts, and rebalancing negative perspectives with future-oriented solutions to the presented problems. Rebalancing the overload of negative information present during pandemics with solutions on how to tackle the pandemic is also important from a psychological perspective, with the ability to increase the psychological need of competence and self-efficacy [58].

Governments might also use the research on incidental exposure to news to position information on platforms and in contexts where the information is likely to be seen, even in passing, especially considering that there are groups of people who use social media as their primary source of news [41]. As both this study and other studies have shown associations of social media use for news consumption regarding the current pandemic and social media use in general with the symptoms of psychopathology, it becomes vitally important to engage readers to both seek and receive fact-checked information on these media. Another possible solution could be attempted by presenting imperative pandemic-related news along with entertainment programs on television, and in the context of entertainment on the internet, previous studies have shown that people are exposed to this information even in passing [41]. Through these strategies designed to tackle intentional and unintentional avoidance, journalists and health-policy makers may provide avoidant individuals with essential life-saving information, and ease added symptoms of psychopathology associated with avoidance of news related to the pandemic.

### Strengths and Limitations

A major strength of this study is that data collection was conducted in a period where pandemic restrictions had just been lifted, and no significant changes to the pandemic protocols in Norway occurred. Although the collected data were not fully representative of the Norwegian population given the oversampling and undersampling of several highlighted subgroups, poststratification weights were calculated and applied to closely match the sample with the true distribution in the population, yielding a representative sample of the Norwegian adult population. The study used a cross-sectional design, which impairs the ability to infer causal effects. Another limitation is using self-reporting of symptoms and inquiring about the presence of a psychiatric diagnosis rather than performing diagnostic interviews. As with traditional sampling techniques involving voluntary participation, potential self-selection of participants may have occurred. However, self-selection was accounted for statistically through the described weighting and raking procedures.

### Conclusion

This study found significant associations between the symptoms of psychopathology and the use of media for obtaining information related to the COVID-19 pandemic. Significant findings for obtaining information through newspapers, social media, and online interactive media were seen across all 3 measures of psychopathology. Avoidance of COVID-19–related information and associations with the symptoms of psychopathology emerged as core findings, with generally higher effect sizes compared with informational attainment. In the age of social media and fast-paced informational streams, presenting up-to-date curated pandemic information is imperative to mitigate the associated detrimental mental health consequences of information dissemination, and efficacious communication is important to hinder viral spread, keep populations up to date on the latest hygienic recommendations, and counteract the effects of infodemic misinformation and news overload. Effectively and accurately conveying information can directly benefit the mental health of citizens.

Further research should be conducted to elucidate the causal mechanisms underlying the associations between media use and the symptoms of psychopathology, and between news avoidance and the symptoms of psychopathology. Public health officials must direct their efforts toward creating better opportunities for individuals to acquire validated information related to the pandemic, nudging them to trusted sources of information, and preventing news overload.

## Appendix

Multimedia Appendix 1 Data for media variables.

Multimedia Appendix 2 Correlation matrix for media variables.

Multimedia Appendix 3 Predictors of anxiety symptoms in the weighted representative sample.

Multimedia Appendix 4 Predictors of depressive symptoms in the weighted representative sample.

Multimedia Appendix 5 Predictors of health anxiety symptoms in the weighted representative sample.

## Abbreviations

GAD-7: Generalized Anxiety Disorder-7
PHQ-9: Public Health Questionnaire-9
VMP: viral mitigation protocol
