# Supplementary material for: Pandemic Information Dissemination and Its Associations With the Symptoms of Mental Distress During the COVID-19 Pandemic: Cross-sectional Study
Source: JMIR Form Res. 2021 Dec 3;5(12):e28239. doi: 10.2196/28239 (PMC8647975; doi:10.2196/28239)
Supplement: Multimedia Appendix 1 [file formative_v5i12e28239_app1.docx]

**Multimedia Appendix 1.** Data for media variables.

|  | Mean | SD | Median | IQR |
| --- | --- | --- | --- | --- |
| Newspapers | 3.34 | 1.64 | 3 | 3 |
| TV | 2.49 | 1.66 | 2 | 3 |
| Social media | 2.14 | 1.85 | 2 | 2 |
| Forums and blogs | 0.96 | 1.21 | 1 | 1 |
| Friends and family | 1.70 | 1.19 | 1 | 1 |
| Others | 1.15 | 1.19 | 1 | 2 |
| Avoidance | 0.86 | 1.23 | 0 | 1 |
| Note. Media variables are values meaning time spent on each media. Avoidance of information is rated on a scale from 1 to 7. | | | | |
