# Supplementary material for: Pandemic Information Dissemination and Its Associations With the Symptoms of Mental Distress During the COVID-19 Pandemic: Cross-sectional Study
Source: JMIR Form Res. 2021 Dec 3;5(12):e28239. doi: 10.2196/28239 (PMC8647975; doi:10.2196/28239)
Supplement: Multimedia Appendix 2 [file formative_v5i12e28239_app2.docx]

**Multimedia Appendix 2.** Correlation matrix for media variables.

|  | Newspapers | TV | Social media | Forums and blogs | Friends and family | Others | Avoidance |
| --- | --- | --- | --- | --- | --- | --- | --- |
| Newspapers | 1.00 | 0.47 | 0.40 | 0.31 | 0.29 | 0.23 | -0.22 |
| TV | 0.47 | 1.00 | 0.31 | 0.22 | 0.24 | 0.17 | -0.17 |
| Social media | 0.40 | 0.31 | 1.00 | 0.43 | 0.45 | 0.23 | -0.06 |
| Forums and blogs | 0.31 | 0.22 | 0.43 | 1.00 | 0.34 | 0.33 | -0.08 |
| Friends and family | 0.29 | 0.24 | 0.45 | 0.34 | 1.00 | 0.28 | -0.01 |
| Others | 0.23 | 0.17 | 0.23 | 0.33 | 0.28 | 1.00 | -0.06 |
| Avoidance | -0.22 | -0.17 | -0.06 | -0.08 | -0.01 | -0.06 | 1.00 |
