# Supplementary material for: Pandemic Information Dissemination and Its Associations With the Symptoms of Mental Distress During the COVID-19 Pandemic: Cross-sectional Study
Source: JMIR Form Res. 2021 Dec 3;5(12):e28239. doi: 10.2196/28239 (PMC8647975; doi:10.2196/28239)
Supplement: Multimedia Appendix 3 [file formative_v5i12e28239_app3.docx]

**Multimedia Appendix 3.** Predictors of anxiety symptoms in the weighted representative sample.

|  | Beta | SE of B | *P* | Part corr, r |
| --- | --- | --- | --- | --- |
| Intercept | 5.42 | 0.38 | *<*.001 | 1.00 |
| Gender^a^ | -0.60 | 0.18 | .0011 | -0.06 |
| Age | -0.06 | 0.01 | *<*.001 | -0.20 |
| Education | -0.18 | 0.09 | .04 | -0.04 |
| Mental health condition | 4.35 | 0.29 | *<*.001 | 0.37 |
| Traditional Media | 0.15 | 0.04 | *<*.001 | 0.08 |
| Online Interactive Media | 0.21 | 0.05 | *<*.001 | 0.10 |
| Friends and family | -0.07 | 0.10 | .52 | -0.01 |
| Others | 0.04 | 0.08 | .60 | 0.01 |
| Avoidance | 0.35 | 0.08 | *<*.001 | 0.10 |
| Note. N = 4921, Adjusted R2 = 0.33 ^a^ Female = 0; Male = 1 | | | | |
