# Supplementary material for: Pandemic Information Dissemination and Its Associations With the Symptoms of Mental Distress During the COVID-19 Pandemic: Cross-sectional Study
Source: JMIR Form Res. 2021 Dec 3;5(12):e28239. doi: 10.2196/28239 (PMC8647975; doi:10.2196/28239)
Supplement: Multimedia Appendix 4 [file formative_v5i12e28239_app4.docx]

**Multimedia Appendix 4.** Predictors of depressive symptoms in the weighted representative sample.

|  | Beta | SE of B | *P* | Part corr, r |
| --- | --- | --- | --- | --- |
| Intercept | 8.75 | 0.53 | *<*.001 | 1.00 |
| Gender^a^ | -0.68 | 0.25 | .005 | -0.05 |
| Age | -0.08 | 0.01 | *<*.001 | -0.18 |
| Education | -0.31 | 0.12 | .007 | -0.05 |
| Mental health condition | 6.43 | 0.39 | *<*.001 | 0.41 |
| Traditional Media | 0.07 | 0.05 | .14 | 0.03 |
| Online Interactive Media | 0.22 | 0.06 | *<*.001 | 0.08 |
| Friends and family | -0.27 | 0.13 | .04 | -0.04 |
| Others | -0.02 | 0.10 | .86 | -0.00 |
| Avoidance | 0.63 | 0.11 | *<*.001 | 0.13 |
| Note. N = 4921, Adjusted R2 = 0.36 ^a^ Female = 0; Male = 1 | | | | |
