# Supplementary material for: Pandemic Information Dissemination and Its Associations With the Symptoms of Mental Distress During the COVID-19 Pandemic: Cross-sectional Study
Source: JMIR Form Res. 2021 Dec 3;5(12):e28239. doi: 10.2196/28239 (PMC8647975; doi:10.2196/28239)
Supplement: Multimedia Appendix 5 [file formative_v5i12e28239_app5.docx]

**Multimedia Appendix 5.** Predictors of health anxiety symptoms in the weighted representative sample.

|  | Beta | SE of B | *P* | Part corr, r |
| --- | --- | --- | --- | --- |
| Intercept | 0.70 | 0.17 | *<*.001 | 1.00 |
| Gender^a^ | -0.15 | 0.09 | .095 | -0.04 |
| Age | 0.00 | 0.00 | .56 | 0.01 |
| Education | -0.18 | 0.04 | *<*.001 | -0.09 |
| Mental health condition | 1.20 | 0.14 | *<*.001 | 0.24 |
| Traditional Media | 0.11 | 0.02 | *<*.001 | 0.14 |
| Online Interactive Media | 0.10 | 0.02 | *<*.001 | 0.10 |
| Friends and family | -0.03 | 0.05 | .55 | -0.02 |
| Others | 0.01 | 0.04 | .78 | 0.01 |
| Avoidance | 0.09 | 0.03 | .007 | 0.06 |
| Note. N = 4921, Adjusted R2 = 0.15 ^a^ Female = 0; Male = 1 | | | | |
